# Decreased Capacity for Sperm Production Induced by Perinatal Bisphenol A Exposure Is Associated with an Increased Inflammatory Response in the Offspring of C57BL/6 Male Mice

**DOI:** 10.3390/ijerph15102158

**Published:** 2018-10-01

**Authors:** Yuan Meng, Ren Lin, Fengjuan Wu, Qi Sun, Lihong Jia

**Affiliations:** Department of Child and Adolescent Health, School of Public Health, China Medical University, Shenyang 110122, China; yuanyuan_max@163.com (Y.M.); 15804066682@163.com (R.L.); 18524430655@163.com (F.W.); sunqi@cmu.edu.cn (Q.S.)

**Keywords:** bisphenol A, sperm production, inflammation response

## Abstract

Many previous studies have indicated the adverse effects of bisphenol A (BPA) on sperm production and quality; however, the mechanisms underlying BPA male reproductive toxicity have yet to be elucidated. The main purpose of this study was to investigate the effect of perinatal exposure to BPA on the spermatogenic capacity of male offspring, and to explore the possible influence of inflammatory responses in BPA reproductive toxicity. Twenty-one pregnant C57BL/6mice were randomly divided into three groups: a control group, a group receiving 0.2 μg/mL (LBPA), and a group receiving 2 μg/mL of BPA (HBPA), all via drinking water from gestational day 6 to the end of lactation. After weaning, one male mouse was randomly selected from each group (*n* = 7/group); these three mice were fed a normal diet and drinking water for 1 month. Levels of serum testosterone (T) and tumor necrosis factor (TNF)-α were then measured in all mice. Sperm count and the proportion of sperm malformation were also determined. The levels of Toll-like receptor 4 (TLR4), nuclear factor (NF)-κB, and aryl hydrocarbon receptor (AhR) protein expression in the testis tissue were determined. Analysis showed that the proportion of sperm malformation increased in the LBPA and HBPA groups (*p* < 0.05). Sperm count significantly decreased only in the HBPA group (*p* < 0.05), while the levels of serum TNF-α increased in the LBPA and HBPA groups (*p* < 0.05). Levels of serum T decreased significantly in the HBPA group, compared with controls (*p* < 0.05). Levels of TLR4 and NF-κB protein expression in the testis were significantly higher in the LBPA and HBPA groups (*p* < 0.05 or *p* < 0.01), while AhR protein expression was higher and seminiferous tubules in the testis showed more damage in the HBPA group compared to controls (*p* < 0.05 and *p* < 0.01, respectively). Our results showed that perinatal exposure to low or high doses of BPA decreased the capacity for spermatogenesis in male offspring, which may be associated with an inflammatory response activated by the TLR4/ NF-κB and AhR signaling pathways in the testis.

## 1. Introduction

There is significant interest in the potential impact of environmental toxicity, such as fungicides, insecticides, and herbicides, on male reproductive potential, with several existing publications reporting reductions in semen quality [1,2,3]. The reproductive toxicity of bisphenol A (BPA) has aroused concern with our widespread use of plastic products. Bisphenol A is a well-known endocrine-disrupting chemical (EDC), and is used primarily to make polycarbonate (PC) plastics and epoxy resins. Bisphenol A -based plastic is clear and tough, and is incorporated into many common consumer goods, including water bottles and coatings on the inside of many food and beverage cans [4]. Therefore, humans are often exposed to BPA in their daily lives. The U.S. Center for Disease Control and Prevention (CDC) has reported measurable levels of BPA in the urine samples of 90% of the U.S. population [5]. It is also noteworthy that BPA has been detected in amniotic fluid, cord blood, and human breast milk, which demonstrates the possibility of BPA being transmitted from the mother to the fetus [6,7]. Increasing evidence also indicates the adverse effects of BPA on the nervous, endocrine, and reproductive systems [8].

At present, most studies relate to the effect of BPA on the reproductive system. Studies in human subjects have consistently demonstrated a relationship between BPA exposure and decreasing fecundity, low sperm count, abnormal sperm function, and increasing morphological abnormalities in spermatozoa [9,10]. However, there are conflicting papers that suggest that BPA exposure promotes spermatogenesis [11]. Gestational BPA exposure can reduce the proportion of elongated spermatids in the seminiferous tubules in pubertal mice [12], impair steroidogenesis in rodents [13], and cause pathological damage to the testicles and ovaries of male and female offspring [14]. However, the precise molecular mechanisms underlying how BPA affects fertility are still poorly understood.

Bisphenol A mimics estrogen and antagonizes androgens because of its molecular structure, which is similar to that of estradiol (E2) [15,16]. Therefore, the mechanism of BPA action may be associated with membrane-bound estrogen receptors, androgen receptors, peroxisome receptor γ, and thyroid receptors [13]. A recent experimental study showed that BPA has estrogenic effects on female reproduction but does not mimic all of the effects of E2 [17]. Bisphenol A could also bind directly to androgen receptors and block the effects of endogenous androgens [18]. The adverse effect of BPA on the reproductive system changes with exposure window, dose, duration, and species or strain. Therefore, it is worth studying the effects of perinatal BPA exposure on reproductive function in offspring and its mechanism of action. This is because many studies of abnormal reproductive function induced by BPA exposure have focused more upon on the adolescent or adulthood periods [19,20].

Recent studies showed positive associations of BPA exposure with proinflammatory cytokines, such as tumor necrosis factor (TNF)-α and interleukin (IL)-6 [21,22]. Nuclear factor-κB (NF-κB) is a ubiquitous multi-directional nuclear transcription factor, representing the confluence of multiple signaling pathways and playing a major role in regulating inflammatory responses. This pathway is important because one of the most important mechanisms of environmental toxic chemicals on adverse health is due to the activation of an inflammatory response. However, few studies have investigated the relationship between abnormal reproductive function and inflammatory responses after BPA exposure. Therefore, we aimed to investigate the effect of perinatal BPA exposure on the capacity of sperm production, as well as inflammation response in male offspring.

## 2. Materials and Methods

### 2.1. Experimental Animals

C57/BL/6 female (20 ± 25 g body weight) and male (23 ± 30 g body weight) mice were purchased from the animal center of China Medical University (license number, SYXL (Liao) 2008–0005). All animals were handled in accordance with the Guidelines for Animal Experimentation issued by the Chinese Association for Laboratory Animal Science. After a one-week adaptation period in a room with a standard temperature (22 ± 2 °C) and illumination (12 h light-dark), females were mated with males. A sperm-positive vaginal smear was taken to indicate the first day of pregnancy. Pregnant mice of normal physiological condition were housed individually and randomly allocated into three groups (*n* = 7/group). Two groups were exposed to BPA (Sigma-Aldrich Chemical Co. St. Louis, MO, USA) by free access to water at levels of 0.2 μg/mL (LBPA) and 2 μg/mL (HBPA) from gestation day 6 until the end of lactation. The third group, as a control group, was given water containing 1% ethanol, the concentration used as vehicle for BPA solution. Maternal food intake and water consumption were measured daily during gestation and lactation. The first day of parturition was recorded as postnatal day 0 (PND0). From PND21, the end of lactation, male offspring were given normal water without BPA and fed with a standard chow diet until PND49. The female offspring were used for other studies. Water consumption was measured daily according to volume reduction in the bottles, and body weight was measured weekly for male offspring. At the end of the experiment (PND49), seven male offspring, belonging to seven different litters, were randomly chosen from each group. After an overnight fasting period, offspring were anesthetized with ether and blood samples were taken from the abdominal aorta. Testis and epididymis were then dissected. The testis tissue was subsequently weighed and the organ coefficients were calculated (organ coefficient = weight of organ/weight of mice). Separated serum and testis were stored at −80 °C until subsequent analysis, and epididymi were used to detect the capacity of sperm production through a specific method [23].

### 2.2. Analysis of Sperm Count and Sperm Abnormality Rate

Two epididymi from each male offspring were placed in 0.5 mL of sodium chloride solution (0.9%, 37 °C). After crushing the tissue to remove debris, 3.5 mL of sodium chloride was added into the sperm suspension. One smear from each sperm suspension was examined with an Olympus light BX63 microscope (Olympus Corporation, Tokyo, Japan) using a 100× objective to evaluate the number of sperm. Two drops of sperm suspension were dropped onto glass slides and then naturally dried at room temperature and fixed with methanol. After 2% eosin staining for 30 min, the morphology of the spermatozoa was observed under an inverted microscope at a magnification of 400× and photographed to calculate the malformation rate. Indicators of sperm deformity included the lack of a hooked head, a big head, a banana shape, a double head and a double tail. In brief, 500 sperm on each glass slide were observed under the microscope from each sperm suspension. We then recorded the number of sperm with abnormal morphology. This allowed us to calculate the sperm malformation proportion.

### 2.3. Histological Change in the Testis

Pathological changes in the testis were examined after hematoxylin and eosin (H&E) staining. Testicular tissue samples were fixed in 10% buffered formalin, dehydrated, and embedded in wax. Serial sections of the tissues were then prepared in an automated microtome at a thickness of 5 µm, stained with H&E, and then assessed with an optical microscope at 200× magnification.

### 2.4. Biochemical Assays

Fasting blood samples were collected from the retro-orbital plexus, and serum was separated by centrifugation. Levels of serum testosterone (T) and TNF-α were determined using a commercially available reagent kit (Enzo Life sciences, Farmingdale, NY, USA; Biosino Biotechnology and Science, Beijing, China) which was used in accordance with the manufacturer’s instructions.

### 2.5. Western Blot Analysis

The protein expression levels of NF-κB, Toll-like receptor 4 (TLR4), and aryl hydrocarbon receptor (AhR) were analyzed by standard Western blotting methods. Testis tissue samples were first homogenized in protein lysate and then centrifuged (1200× *g*, 4 °C) for 15 min. Protein concentration was then determined using a bicinchonininc acid (BCA) protein assay kit (Beijing Ding Guo Chang Sheng Biotechnology, Beijing, China). Subsequently, samples containing 50 μg of total protein were mixed with equal volumes of 5× sodium dodecyl sulfate (SDS)-polyacrylamide gel loading buffer and incubated at 95 °C for 5 min. Ten microliters of each sample were then loaded into the wells of 10% SDS-acrylamide gels and the proteins were separated and electrophoretically transferred onto polyvinylidene fluoride (PVDF) membranes for 1.5 h at 100 V. Blots were incubated for 2 h at 4 °C with tris buffered saline (TBS) containing 0.1% Tween-20 and 5% nonfat milk (TBSTM), followed by incubation with specific primary antibodies including: NF-κB (1:1000, #8242, Cell Signaling Biotechnology, Danvers, MA, USA), TLR4 (1:200, sc-293072, Cell Signaling Biotechnology), and AhR (1:1000, NB100-2289, Novus Biological, Littleton, CO, USA) in TBSTM at 4 °C overnight. Membranes were then incubated for 2 h with HRP-conjugated anti-rabbit IgG antibody (1:5000, abs20002A, Absin, China) or HRP-conjugated anti-mouse IgG antibody (1:5000, abs20001A, Absin, China). An antibody against glyceraldehyde-3-phosphate dehydrogenase (GAPDH) was used as an internal reference. Immunosuppressant bands were finally visualized with a chemiluminescence kit (NC15079, Thermo Fisher Scientific, Waltham, MA, USA) and quantified using image analysis software (Scion Image, version Beta 4.0.3 Thermo Fisher Scientific, Waltham, MA, USA). Four samples from each group were analyzed.

### 2.6. Statistical Analysis

All analyses were performed using SPSS 20.0 software (SPSS, Inc., Chicago, IL, USA). Data were expressed as means ± standard errors (SE). Differences between groups were analyzed by least-significan difference LSD post-hoc tests after one-way analysis of variance (ANOVA). *p* < 0.05 was considered to be statistically significant.

## 3. Results

### 3.1. Effect of Perinatal Bisphenol A Exposurure on Body Weight and Food, Water Intake in Maternal Mice and Their Offspring

**Figure 1:**
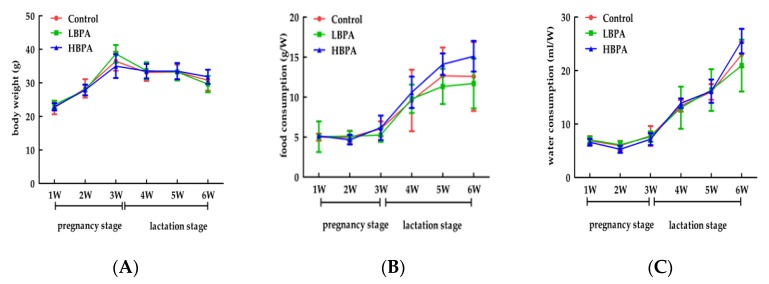
The effects of perinatal bisphenol A (BPA) exposure on maternal body weight, food intake, and water consumption. Pregnant mice (*n* = 7/group) were exposed to ethanol or BPA in water at levels of 0.2 μg/mL (LBPA) or 2 μg/mL (HBPA) from gestational day 6 through to the end of lactation. Changes in mean body weight (**A**), amount of food intake (**B**), and water intake (**C**) were measured during pregnancy. Values represent the mean ± standard error (SE) and were analyzed by one-way analysis of variance (ANOVA).

Mean body weight, food intake, and consumption of drinking water during pregnancy showed no significant differences when compared between maternal mice exposed to BPA and controls (*p* > 0.05, Figure 1). Similarly, there were no significant differences in terms of mean body weight, food intake, or drinking water consumption in male offspring exposed to BPA compared with controls after weaning (*p* > 0.05).

### 3.2. The Effect of Perinatal Bisphenol A Exposure on Sperm Production Capacity in Male Offspring

Pregnant mice (*n* = 7/group) were exposed to ethanol or BPA in water at levels of 0.2 μg/mL or 2 μg/mL from gestational day 6 through to the end of lactation. Changes in mean body weight (A), food intake (B), and water consumption (C) were measured on PND49. Values represent the mean ± standard error (SE) (*n* = 7, one from each group) and were analyzed by one-way analysis of variance (ANOVA).

**Figure 2:**
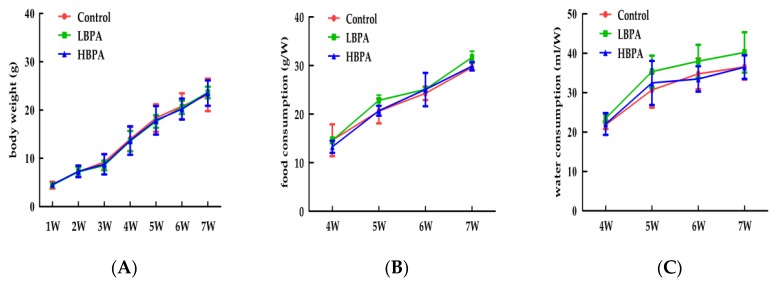
Effects of perinatal BPA exposure on mean body weight, food intake, and water intake in male offspring mice. Changes in mean body weight (**A**), food intake (**B**), and water consumption (**C**).

**Figure 3:**
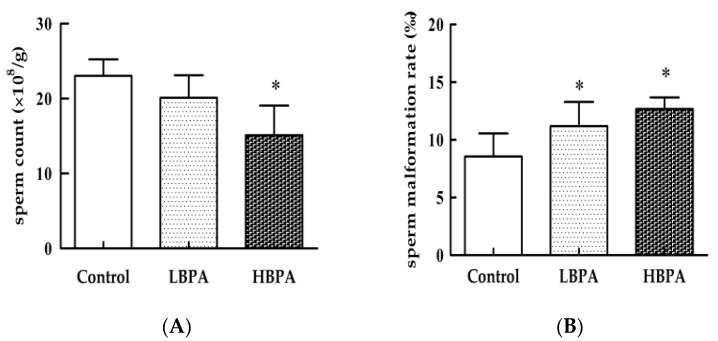
Effects of perinatal BPA exposure on sperm count (**A**) and sperm malformation rate (**B**) in the epididymi of male offspring. Data are reported as means ± SE (*n* = 7, one from each group) and were analyzed by one-way ANOVA. * *p* < 0.05 compared with controls.

There were no statistically significant (*p* > 0.05) differences in terms of the weight or coefficient of the testis and epididymis when compared between the LBPA group and the HBPA group (Table 1). Results relating to spermatogenic ability are shown in Figure 2. There was no significant difference in sperm count between the LBPA and the control groups (*p* > 0.05). However, the rate of sperm malformation was significantly higher (*p* < 0.05) in the LBPA group. In the HBPA group, there was a significantly lower sperm count and a significantly higher rate of sperm malformation (*p* < 0.05, Figure 3).

**Figure 4:**
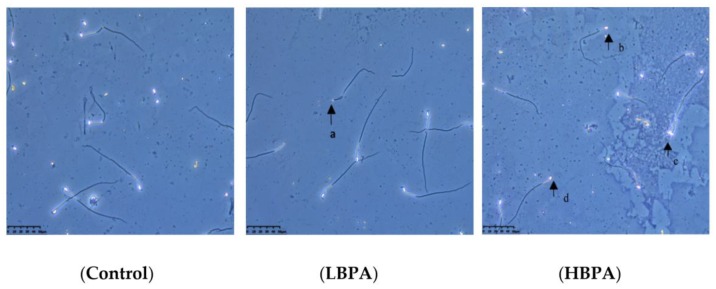
Effects of perinatal BPA exposure on sperm morphology in male offspring. (**a**) microcephalus malformation; (**b**,**d**) hookless malformation; (**c**) dicephaly. Magnification: Control, LBPA, and HBPA, 400×.

When observed under an inverted microscope, the sperm from male offspring in the control group showed normal morphology and structural integrity (Figure 4). The sperm from male offspring in the LBPA group showed deformities, mainly consisting of microcephalus and the lack of a hooked head. Sperm from male offspring in the HBPA group exhibited diplocephaly; furthermore, there was an increased incidence of microcephalus and hookless malformations.

**Figure 5:**
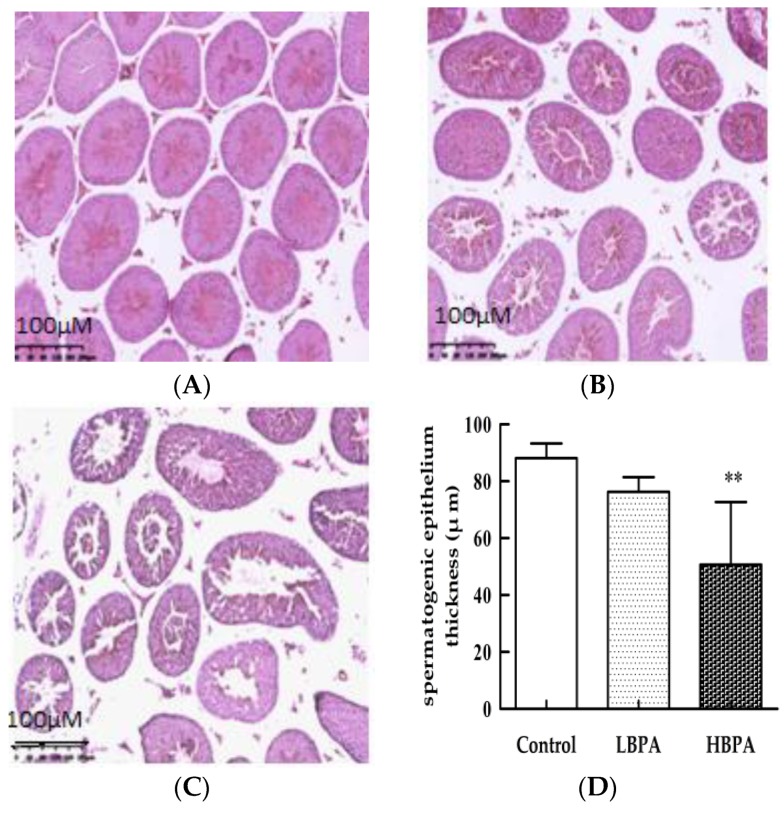
Effects of perinatal BPA exposure on pathological damage in the testis tissue of male offspring. Histology of control mice (**A**), 0.2 μg/mL BPA (**B**), and 2 μg/mL BPA (**C**). Semi-quantitative analysis of testicular spermatogenic epithelium thickness by hematoxylin and eosin (H&E) staining (**D**). ** *p* < 0.01 compared with controls. Magnification: **A**–**C**, 200×.

Results showed that the seminiferous tubules in the testis of mice in the control group were arranged neatly, and that in cross-section, they were round or oval under the light microscope. At all levels, the spermatogenic cells were arranged in order on the wall of the tubules; the structure was complete, and a large number of immature sperm cells were visible (Figure 5A). In the LBPA group, some of the seminiferous tubules were atrophied; spermatogenic cells were disordered, and the number of sperms in the lumen was reduced (Figure 5B). In the HBPA group, most of the seminiferous tubules were atrophied, the number of spermatogenic cells was reduced; furthermore, sperm cells showed disorder, with most showing degeneration and edema. Sperm count was significantly lower (*p* < 0.05) and the lumen space was larger. The shedding of immature spermatogenic cells was observed in the lumen (Figure 5C). The thickness of the seminiferous epithelial cells was significantly reduced in the HBPA group compared with controls (*p* < 0.01, Figure 5D).

### 3.3. The Effect of Perinatal Exposure to Bisphenol A on Levels of Serum Testosterone and Tumor Necrosis Factor-α in Male Offspring

The effects of perinatal exposure to BPA on levels of serum testosterone and TNF-α are shown in Table 2. The levels of serum testosterone were significantly lower only in the HBPA group compared with controls (*p* < 0.01). However, serum TNF-α levels were significantly higher in BPA-exposed male offspring than that in controls (*p* < 0.05).

### 3.4. The Effect of Perinatal Exposure to Bisphenol A on Inflammatory Factor-Related Protein Expression in Testis Tissue from Male Offspring

**Figure 6:**
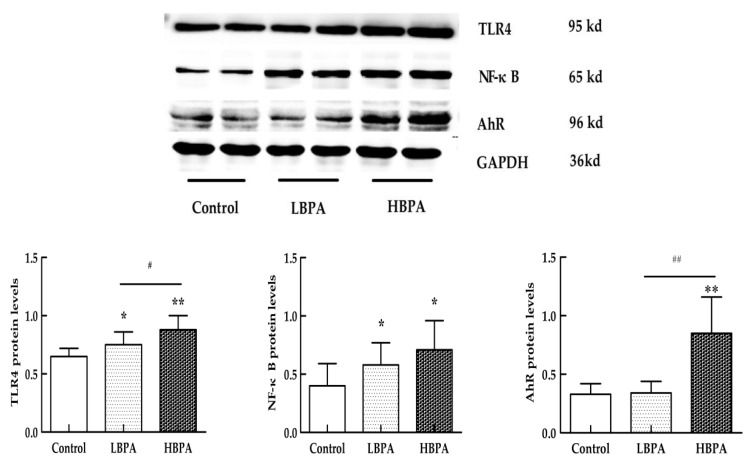
Effects of perinatal BPA exposure on the levels of Toll-like receptor 4 (TLR4), nuclear factor (NF)-κB, and aryl hydrocarbon receptor (AhR) protein expression in testis tissue in male offspring. Pregnant mice (*n* = 7/group) were exposed to ethanol or BPA in water at levels of 0.2 μg/mL or 2 μg/mL from gestational day 6 through to the end of lactation. The levels of TLR4, NF-κB, and AhR protein expression in testis tissue were measured by Western blotting in male offspring. Values represent the means ± SE (*n* = 7, one from each group). * *p <* 0.05 and ** *p <* 0.01 compared with controls. ^#^ *p <* 0.05 and ^##^ *p <* 0.05 compared with the LBPA group.

The levels of TLR4 and NF-κB protein expression were significantly higher in both the LBPA and HBPA groups (*p* < 0.05 or *p* < 0.01), but there was only a significantly higher level of AhR protein expression in the HBPA group compared with controls (*p* < 0.01, Figure 6).

## 4. Discussion

Studying the effects of BPA upon reproductive function has become very important because BPA mimics estrogen and antagonizes androgen. However, the mechanisms underlying the reproductive toxicity of BPA are not clear. Because the effects of BPA upon health changes with dose, time, window of exposure, duration, and the species or strains investigated, we used pregnant C57BL/6J mice and selected 0.2 μg/mL and 2 μg/mL BPA to represent lower and higher doses of BPA exposure, respectively. The low doses of BPA we designed here were based on the United States Environmental Protection Agency (EPA) standards, in which the lowest observable adverse effect level (LOAEL) of BPA was determined to be 50 μg BPA/kg body weight /day [19]. Our results showed that the amount of water consumed was 7.86 ± 0.91 mL in the LBPA group during pregnancy. Mean levels of BPA consumed daily by pregnant females were approximately 1.57 μg BPA/dam during gestation. The body weight of gestating dams was approximately 29.84 ± 6.71 g in the LBPA group before parturition; thus, the levels of BPA exposure were approximately 52.68 μg/kg BW/day in dams from the LBPA group during pregnancy. Because the mice may spill some water during the drinking process, the mean daily BPA exposure of pregnant mice may be less than that observed in our study. In our study, the high dose was 10 times higher than the low dose. Our results indicated that the mean daily volume of water consumed in BPA-exposed dams was not significantly different when compared with controls during pregnancy.

Our results also showed that there were adverse effects of both low and high doses of BPA-exposed dams on sperm production ability in their male offspring. In the LBPA group, the rate of sperm malformation was obviously higher than that in the control group. The sperm appeared to have diplocephaly, and the number of sperm with microcephalus and hookless malformation was significantly increased in male offspring exposed to the higher dose of BPA. Moreover, the rate of sperm malformation increased, and the sperm count decreased, with increasing BPA dose. Furthermore, testicular tissue showed differing degrees of pathological damage in offspring exposed to BPA, and the seminiferous epithelial cells were obviously thinner in the HBPA group compared with controls. Shiet al. recently reported that pathological damage was incurred by the testicles and ovaries of male and female offspring after perinatal exposure to BPA at levels of 50, 500, and 2500 mg/kg [14]. Xieet al. further reported spermatogenic dysfunction accompanied by abnormal spermatogenic cell proliferation and differentiation in mice exposed to 0.01 mg/kg BPA by subcutaneous injection [24]. In another study, exposure to 200 μg/kg of BPA by intragastric administration inhibited the spermatogenic function of 9-week-old Wistar rats [20]. Therefore, previous studies have shown that BPA clearly has adverse effects on spermatogenic function in rodents, which is consistent with our present results. Our study also indicated that there was a decreasing trend of spermatogenesis ability, and an increasing rate of sperm malformation and pathological damage, in testicular tissue in male offspring exposed to 0.2 μg/mL BPA, although this was not statistically different when compared with controls. The testis is mainly composed of seminiferous tubules and interstitial tissues, and secretes androgens. Spermatogenic cells in the epithelium of the seminiferous tubules develop into spermatozoa step by step, and the rate of spermatogenesis decreases in response to interference in the differentiation and development of spermatogonia. Studies have also revealed that 10 μM of BPA increased levels of apoptosis in the reproductive cells of human testes in vitro [25].

Recent studies reported adverse health effects of BPA on the development of the brain neural system [26], abnormal glucose and lipid metabolism, and carcinogenesis [27,28], which may be related to an increased inflammatory response [20,21]. However, few studies have focused on the inflammatory response arising from BPA reproductive toxicity. Our results showed that the level of serumTNF-αwas significantly higher, and the levels of nuclear factor-κB (NF-κB) andTLR4protein in the testis increased in male offspring exposed to BPA compared with controls. Collectively, these data indicate that perinatal BPA exposure could increase the inflammatory response in the testis of male offspring via the TLR4/NF-κB signaling pathway. The expression of theAhR protein in the testis also increased in offspring exposed to 2 μg/mL BPA but not 0.2 μg/mL of BPA. As a ubiquitous nuclear transcription factor, NF-κB has multiple regulatory functions; it is the convergence point of multiple signaling pathways and plays an important role in regulating the inflammatory response. Studies have shown that BPA binds to TLR cytokines in adipocytes, and increases the levels of inflammatory cytokines by activating the JNK/STAT3/NF-κB signal pathway, eventually leading to endocrine disruption [29]. Izumi et al. reported that the adverse effects of BPA on sperm autogenesis in the Sertoli cells of rat testis were possibly regulated via increasing levels of inflammatory factors, such as IL-1β and IL-6 [30]. Ogo et al. further showed that exposure to 200 μg/mL of BPA from PND36 to PND66 increased the expression of IL-6 in the epididymis of male Wistar rats [31]. Our current results implied that the adverse effects of perinatal BPA exposure on spermatogenesis might be related to the activation of an inflammatory response in the testis of male offspring.

TheAhR is a cytoplasmic transcription factor, and has a central role not only in the differentiation and maturation of many tissues, but also in the toxicological metabolism of cells via the activation of metabolic enzymes [32]. According to recent studies, the AhR is essential for the differentiation and activation of T helper 17 (Th17) cells [33]. It is well-known that Th17 cells have a central role in the development of inflammation, which is crucial in the defense against pathogens. This data shows that the AhR is closely related to the inflammatory response pathway. Population studies further suggest that the AhR may serve as a potential biomarker of endocrine-disrupting effects on reproductive health [34,35]. In addition, experimental studies found that BPA inhibits cultured mouse ovarian follicle growth, at least in part via the AhR signaling pathway [36]. As a transcription factor, the AhR modulates immune reactions and causes immune toxicity through transcriptional changes [37]. Vogel also showed that the high expression of AhR had a positive correlation with NF-κB in breast cancer tissues [38]. Therefore, we speculate that the reduced levels of spermatogenesis induced by perinatal BPA exposure may be associated with the activation of the inflammatory response by the AhR and TLR4/NF-κB signaling pathways.

In summary, our results indicate that perinatal BPA exposure can decrease spermatogenesis ability and increase the rate of sperm deformity in male offspring, which may be associated with the activation of an inflammatory response involving the AhR and TLR4/NF-κB signaling pathways.

## 5. Conclusions

Pregnancy and lactation are critical periods for reproductive development in offspring. Exposure to BPA during this period will interfere with the development and function of reproductive systems, as well as activate an inflammatory response. Our study implied that the cause of abnormal reproductive function may be related to BPA exposure in early development in humans.

## Figures and Tables

**Table 1:** Effects of perinatal BPA exposure on the weight and organ coefficient (%) of testis and epididymal tissue (g) in male offspring. Pregnant mice (*n* = 7/group) were exposed to ethanol or BPA in water at levels of 0.2 μg/mL (LBPA) or 2 μg/mL (HBPA) from gestational day 6 through to the end of lactation. Values represent the mean ±SE (*n* = 7, one from each group) and were analyzed by one-way ANOVA.

Group	Testis		Epididymis	
	Weight (g)	Coefficient (%)	Weight (g)	Coefficient (%)
Control	0.15 ± 0.03	0.65 ± 0.06	0.08 ± 0.02	0.37 ± 0.03
LBPA	0.16 ± 0.02	0.70 ± 0.09	0.09 ± 0.01	0.39 ± 0.05
HBPA	0.16 ± 0.03	0.68 ± 0.08	0.08 ± 0.01	0.35 ± 0.04

**Table 2:** Effects of perinatal BPA exposure on serum testosterone and tumor necrosis factor (TNF)-α levels in male offspring. Pregnant mice (*n* = 7/group) were exposed to ethanol or BPA in water at levels of 0.2 μg/mL or 2 μg/mL from gestational day 6 through to the end of lactation. Values represent the means ±SE (*n* = 7, one from each group).

Group	*n*	Testosterone (ng/mL)	TNF-α (ng/L)
Control	7	13.47 ± 1.12	3.31 ± 0.58
LBPA	7	12.93 ± 0.98	4.26 ± 0.35 *
HBPA	7	10.69 ± 1.04 **	4.46 ± 0.37 *

Note: * *p* < 0.05 and ** *p* < 0.01 compared with the control group.
